# Invasive Coronary Imaging Assessment for Cardiac Allograft Vasculopathy: State-of-the-Art Review

**DOI:** 10.1016/j.jscai.2022.100344

**Published:** 2022-05-13

**Authors:** Negeen Shahandeh, Kuninobu Kashiyama, Yasuhiro Honda, Ali Nsair, Ziad A. Ali, Jonathan M. Tobis, William F. Fearon, Rushi V. Parikh

**Affiliations:** a Division of Cardiology, University of California Los Angeles, Los Angeles, California; b Division of Cardiovascular Medicine, Stanford University, Stanford, California; c DeMatteis Cardiovascular Institute, St Francis Hospital & Heart Center, Roslyn, New York; d Cardiovascular Research Foundation, New York, New York; e Division of Cardiovascular Medicine, Stanford University and VA Palo Alto Health Care Systems, Stanford, California

**Keywords:** Cardiac allograft vasculopathy, heart transplantation, intravascular imaging

## Abstract

Heart transplantation is the standard of care treatment for end-stage heart failure. Therapeutic advances including enhanced immunosuppression and aggressive infectious prophylaxis have led to increased life-expectancy following transplantation; however, cardiac allograft vasculopathy (CAV) remains a leading cause of morbidity and mortality. Although coronary angiography is the current guideline-recommended diagnostic modality for invasive CAV screening, it is limited in its ability to detect early and/or diffuse disease. Efforts to improve outcomes for heart transplant recipients with CAV have focused on developing diagnostic tools with greater sensitivity to capture early CAV in order to better understand the pathobiology and implement treatment to slow disease progression sooner after transplant. The contemporary invasive imaging armamentarium for CAV surveillance includes coronary angiography, intravascular ultrasound, and newer technologies including optical coherence tomography and near-infrared spectroscopy. The present review outlines the use of and data in support of these imaging platforms in the CAV arena and highlights the potential advantages and limitations of each of these modalities.

## Highlights

• Invasive imaging is essential for early diagnosis of cardiac allograft vasculopathy.
• Angiography and intravascular ultrasound are mainstays supported by prognostic data.
• Invasive imaging may shed light on the pathobiology of cardiac allograft vasculopathy.

## Central Illustration

**Figure:**
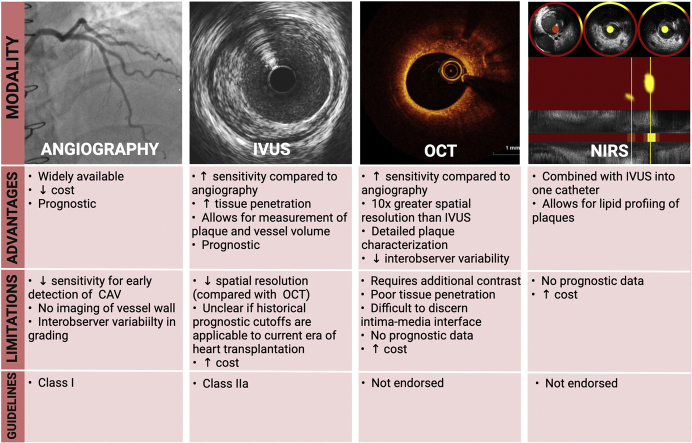

## Introduction

Heart transplantation remains the gold standard treatment for patients with end-stage heart failure. Since the first human-to-human heart transplant was performed in 1967, the field has grown substantially, and today nearly 6000 heart transplants are performed annually.^1^, ^2^, ^3^ Scientific advances have led to increased survival among heart transplant recipients, reflected by a median life expectancy of 12.5 ​years in the contemporary era.^3^^,^^4^ However, despite this progress, cardiac allograft vasculopathy (CAV) has been referred to as the “Achilles’ heel” of long-term survival.^2^

CAV is a complex immune and inflammatory-mediated disease characterized by diffuse intimal hyperplasia and negative remodeling, dual processes that cause accelerated fibroproliferative disease of the allograft coronary circulation.^5^ Alloantigen and foreign human leukocyte antigen presentation by host immune cells trigger an inflammatory cascade that perpetuates inflammation and fibrosis, ultimately leading to early smooth muscle proliferation and matrix deposition in the intima. Chronic vascular injury and endothelial dysfunction result in progressive, diffuse concentric intimal hyperplasia. Concomitant increases in medial tone and adventitial fibrosis lead to negative remodeling, and over time, these processes result in epicardial and microvascular luminal compromise, eventually manifesting as silent myocardial infarction, graft failure, and/or sudden cardiac death.^5^, ^6^, ^7^, ^8^, ^9^

CAV is a leading cause of death in heart transplant recipients, accounting for approximately 10% of deaths at 3 ​years after transplant.^4^ Patients with rapidly progressive CAV at 1 ​year after transplantation have higher rates of death and/or retransplantation at 5 ​years.^10^^,^^11^ Strategies for minimizing the risk of developing CAV include the use of statins, preventing and treating acute rejection with immunosuppressive agents, and aggressive infectious prophylaxis.^7^ In addition to preventing CAV, the use of statins and mammalian target of rapamycin inhibitors has also been demonstrated to decrease rejection and reduce long-term mortality.^12^^,^^13^ Unfortunately, these measures have had a minimal effect on the 5- and 10-year incidence of CAV over the past 20 ​years (32%-30% and 52%-49%, respectively).^4^ Additionally, the 5-year mortality rate after a diagnosis of CAV within 3 ​years of transplant has only marginally improved over this time (28%-22%).^4^ These data highlight that the care of a heart transplant recipient in the contemporary era is greatly limited by the need for more sensitive tools to detect early CAV so that effective treatment to prevent and/or slow its progression may be instituted sooner after transplant. These tools may also provide new insights into the pathobiology of CAV and risk factors for its development that lead to identification and testing of novel therapeutic targets.

Both noninvasive and invasive imaging modalities may be used to screen for CAV. Noninvasive tests such as dobutamine stress echocardiography, coronary computed tomography angiography, and single-photon emission computed tomography have all been studied and found to have a high specificity for detecting angiographic CAV. However, noninvasive modalities lack sensitivity in detecting early CAV, particularly when compared to intravascular imaging, as they cannot assess the vessel wall.^14^, ^15^, ^16^ Because heart transplant recipients often do not develop angina due to denervation of the allograft, CAV can progress to later stages and result in graft failure before patients develop symptoms and/or before it is detectable by noninvasive imaging studies. Consequently, routine invasive imaging to screen for CAV is common in heart transplant recipients.^6^ This report will provide an up-to-date review of the invasive imaging modalities available for use in CAV screening and expand on prior reports by focusing on newer technologies and their role in elucidating the pathobiology of CAV.

## Guideline-recommended invasive imaging modalities for CAV surveillance

### Coronary angiography

**Figure 1:**
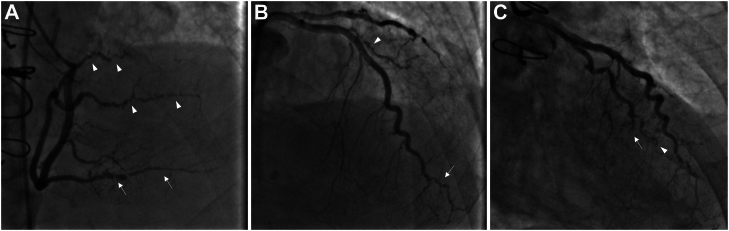
Severe angiographic CAV coronary angiogram demonstrating ISHLT CAV_3_. (**A**) Severe stenoses and distal pruning of the right coronary posterior descending artery (arrows) and posterolateral branches (arrowheads). (**B**) Severe disease and pruning of the distal left anterior descending coronary artery (arrow) and diagonal branch (arrowhead). (**C**) Chronic total occlusion of the distal left circumflex artery (arrow) and severe distal pruning of the obtuse marginal branch (arrowhead). CAV, cardiac allograft vasculopathy; ISHLT, International Society for Heart and Lung Transplantation.

Coronary angiography has traditionally been viewed as the gold standard for the diagnosis and screening of CAV and carries a class I recommendation for post-transplant surveillance.^17^ Several studies have demonstrated the prognostic impact of angiographic CAV. In 1 analysis, patients with angiographic evidence of CAV were noted to have a 19% likelihood of progression to severe CAV within 5 ​years. Furthermore, those with severe CAV had a 50% risk of death or retransplantation at 5 ​years.^18^ The International Society for Heart and Lung Transplantation (ISHLT) has created a standardized grading system for angiographic CAV. The categories of CAV_0_ (nonsignificant), CAV_1_ (mild), CAV_2_ (moderate), and CAV_3_ (severe) disease are designated based on the severity of angiographic stenoses as well as the presence of allograft dysfunction (Table 1).^19^ A subsequent retrospective study using the ISHLT grading system found that CAV_2_ and CAV_3_ were associated with a greater risk of major adverse cardiovascular events (MACEs) than CAV_0_ and CAV_1_.^20^ Accelerated severe angiographic CAV is depicted in Figure 1.

**Table 1:** ISHLT grading classification for CAV.

CAV grade	Angiographic appearance	Allograft function
CAV_0_ (not significant)	No detectable lesion	Normal
CAV_1_ (mild)	LM ​<50%, or	Normal
	Primary vessel lesion <70%, or	
	Branch stenosis <70%	
CAV_2_ (moderate)	LM <50%, and	Normal
	Single primary vessel lesion ≥70%, or	
	Isolated branch stenosis in 2 systems ≥70%	
CAV_3_ (severe)	LM ​≥ ​50%, or	Normal or abnormal
	At least 2 primary vessel lesions ≥70%, or	Normal or abnormal
	Branch stenoses in all 3 systems ≥70%, or	Normal or abnormal
	CAV_1_ or CAV_2_ with allograft dysfunction	Abnormal

CAV, cardiac allograft vasculopathy; ISHLT, International Society for Heart and Lung Transplantation; LM, left main.

Adapted from the 2010 ISHLT, consensus statement for standardized nomenclature of CAV19.

Although angiography is a widely available diagnostic modality and provides valuable prognostic data, there are limitations regarding its sensitivity for detecting early CAV. In 1 study, the sensitivity and specificity of coronary angiography for diagnosing CAV were 42% and 95%, respectively, compared with intravascular ultrasound (IVUS).^21^ Angiography assesses the lumen of epicardial coronary arteries but does not provide imaging of the vessel wall. Early accelerated CAV is more likely to result in diffuse intimal hyperplasia with concentric epicardial disease and negative remodeling and thus may not be recognized on angiography. Indeed, patients may not develop focal obstructive stenoses that can be visualized angiographically until late in the disease process.

### Intravascular ultrasound

**Figure 2:**
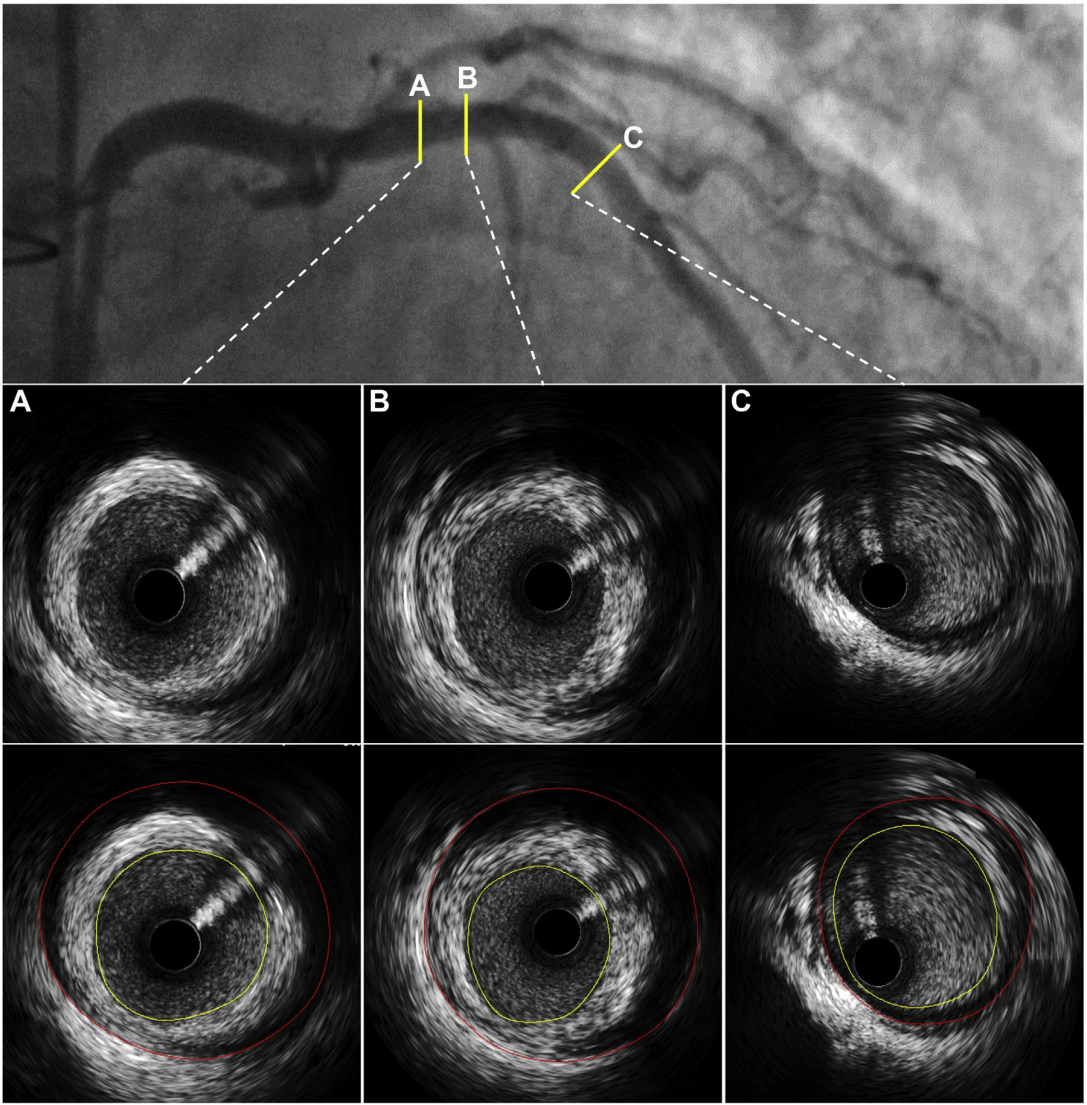
Discordance of coronary angiography and IVUS. The LAD appears to have minimal disease on angiography. IVUS reveals moderate concentric plaque (maximum plaque burden: 71%) in the proximal segment (panels **A** and **B**), as well as mild plaque in the mid segment (panel **C**). IVUS, intravascular ultrasound; LAD, left anterior descending.

Since the 1990s, IVUS has increasingly been used as an adjunct to coronary angiography following heart transplantation, as it provides greater sensitivity in screening for early-onset CAV. Through cross-sectional imaging of the lumen and vessel wall, IVUS can detect early intimal thickening and negative remodeling that is often not yet detectable by conventional coronary angiography (Figure 2). Based on prior pathology studies, an intimal thickness <0.3 ​mm has historically been designated as the upper limit of normal.^22^ A seminal 1992 study established the diagnostic sensitivity of IVUS by demonstrating that 70% of heart transplant recipients had appreciable intimal thickening at 1 ​year despite normal angiograms. These findings led to the development of the Stanford classification system, which uses the degree of intimal thickness and arc, both measured by IVUS, to grade the severity of CAV (Table 2).^23^

**Table 2:** Stanford classification for CAV

	Class I minimal	Class II mild	Class III moderate	Class IV severe
Intimal thickness	<0.3 ​mm	<0.3 ​mm	0.3-0.5 ​mm	>1.0 ​mm
or			or	or
Extent of plaque, arc	<180°	>180°	>0.5 ​mm, <180°	>0.5 ​mm, <180°

CAV, cardiac allograft vasculopathy.

Adapted from the study by St Goar et al.^23^

There are robust data that IVUS-based findings are associated with clinical outcomes in heart transplant patients and have important prognostic implications. In 1 of the first studies to demonstrate the prognostic impact of IVUS in the heart transplant population, a nearly 10-fold increase in the incidence of sudden cardiac death, myocardial infarction, or need for coronary revascularization was noted in those with severe intimal thickening (>0.5 ​mm) compared with those with nonsevere disease over a 4-year follow-up period.^24^ Shortly thereafter, another study found that a mean intimal thickness >0.3 ​mm was significantly associated with decreased 4-year actuarial overall survival (73% vs 96%; *P* ​= ​.005), cardiac survival (79% vs 96%; *P* ​= ​.005), and freedom from cardiac mortality and retransplantation (74% vs 98%; *P* ​< ​.0001).^25^ Notably, in both these studies, the majority of patients had evidence of intimal thickening despite normal coronary angiograms.

Subsequent landmark studies showed that the progression of intimal thickening over the first year after transplant was a key prognostic metric. In a 2005 multicenter study, an increase of ≥0.5 ​mm in maximal intimal thickness (MIT) from baseline to 1 ​year after transplant was associated with increased mortality (20.9% vs 5.9%; *P* ​= ​.025), nonfatal MACE (45.8% vs 16.8%; *P* ​= ​.001), and development of angiographic CAV within 5 ​years (65.2% vs 32.6%; *P* ​= ​.004).^10^ These findings were corroborated by a single-center study that reported similar findings over a 6-year follow-up period.^11^ Together, these studies are the basis for the class IIa guideline recommendation for IVUS and clinical practice of augmenting immunosuppressive therapy in patients exhibiting a change in MIT ≥0.5 ​mm at 1 ​year.^17^ An important caveat of these studies is that they were conducted during an era when 1) immunosuppressive regimens typically included cyclosporine and azathioprine, both of which are less commonly used now in favor of tacrolimus and mycophenolate, and 2) statins and proliferation signal inhibitors (eg, sirolimus), both of which are now known to slow the progression of CAV, had yet to be widely adopted.^26^^,^^27^ Rapid evolution of post-transplant management over the past few decades may affect the validity of using historically established IVUS parameters for prognostication in modern-day patient populations. Indeed, 1 recent study reported that a change in MIT ≥0.5 ​mm at 1 ​year failed to predict long-term clinical outcomes, raising the possibility that early intimal thickening assessed by IVUS may no longer be sufficiently sensitive in predicting late MACE.^28^ However, another contemporaneous study found that a change in MIT ≥0.35 ​mm from 1 ​year to 5 ​years after transplant was associated with both MACE and cardiovascular mortality.^29^

**Figure 3:**
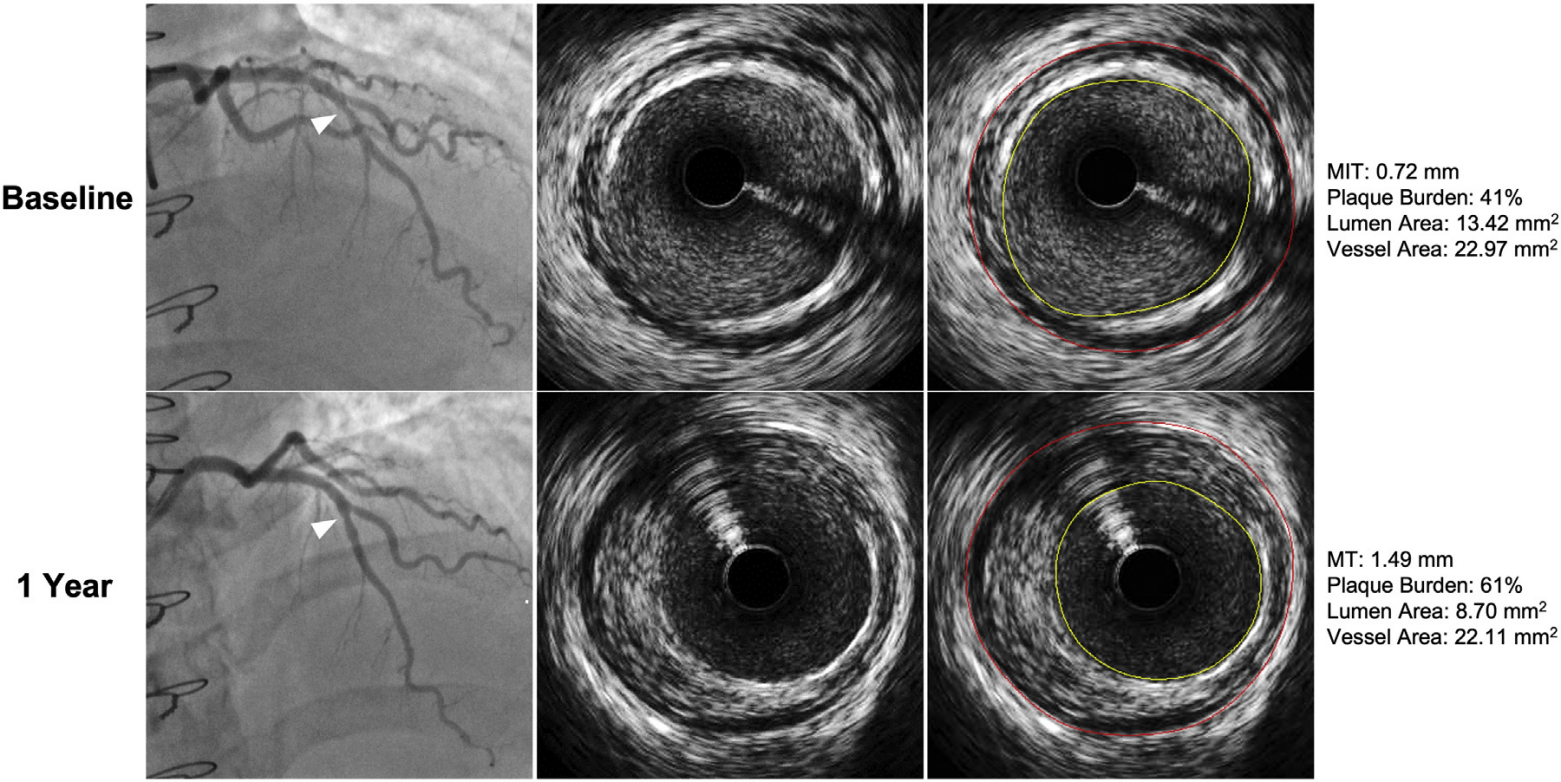
Paradoxical remodeling demonstrated by IVUS 1 ​year after heart transplantation. Serial IVUS cross-sections in a mild stenosis (arrowhead) of the LAD obtained at baseline (upper panels) and 1 ​year (lower panels) after heart transplantation. Maximal intimal thickness (MIT) increased by 0.77 ​mm in a year without compensatory (adaptive) vessel remodeling (ie, paradoxical remodeling). IVUS, intravascular ultrasound; LAD, left anterior descending.

IVUS interrogation can also be used to perform a 3-dimensional (3D) volumetric analysis, which provides greater accuracy in detecting smaller changes in the vessel wall and thus a more precise assessment of disease burden. In 1 study, 3D IVUS was used to characterize the extent of vessel remodeling in 100 heart transplant patients. The authors demonstrated that the combination of increased intimal volume and decreased vessel volume (ie, negative remodeling) at 1 ​year was significantly associated with acute cellular rejection as well as increased risk of mortality and retransplantation over a median follow-up period of 4.7 ​years.^28^ Paradoxical remodeling is often due to inadequate compensatory coronary dilation in response to increases in intimal thickening early in the post-transplant course (Figure 3) and is subsequently followed by a decrease in vessel volume as the intimal volume continues to increase (ie, negative remodeling). Indeed, multiple IVUS studies to date have demonstrated that negative remodeling is a key hallmark of CAV.^5^^,^^8^^,^^9^^,^^30^

Given concerns about the validity of applying historical cutoffs for MIT to patients in the contemporary era of heart transplantation, several recent studies have focused on the use of alternative IVUS parameters for prognostication. For example, 1 study evaluated the association between progression of attenuated-signal plaque, an IVUS marker of necrotic core or lipid pool within plaque, and long-term mortality after heart transplantation. Interestingly, attenuated-signal plaque progression at 1 ​year after transplant was significantly associated with the occurrence of acute cellular rejection (grade ≥2R) and increased long-term mortality and retransplantation.^31^ In another study, heart transplant patients with progression of periarterial neovascularization on IVUS over the first year after transplantation had a greater increase in MIT, a higher incidence of acute cellular rejection, and reduced long-term survival than their counterparts without progression.^32^

Although IVUS appears to have supplanted coronary angiography as the gold standard for early detection of CAV in the contemporary era, its limitations should be considered. First, until the recent introduction of 60-MHz catheters with more rapid catheter pullback speeds, image resolution and acquisition time were notable limitations. Second, IVUS interrogation is typically limited to the left anterior descending (LAD) due to cost, time, and risk considerations including additional contrast use and increased risk of procedural complications with multivessel assessment. Although a pan-coronary approach would provide greater sensitivity, it is not known whether this would translate to additional prognostic or clinical benefits. Third, 3D volumetric analysis is very time-consuming and not practical for routine clinical use. Finally, updated studies evaluating the prognostic implications of IVUS measurements incorporating both intimal thickening and vessel remodeling as well as historical cutoffs in the current era of transplant cardiology are lacking.

## Newer invasive imaging platforms for CAV detection

### Optical coherence tomography

Over the past decade, optical coherence tomography (OCT) has emerged as a potential tool for CAV screening. First available in 2008, OCT uses near-infrared wavelengths of light to image the coronary vessel wall and provides approximately 10-fold greater spatial resolution than IVUS, resulting in near-histologic visualization.^26^^,^^33^ In an early study comparing OCT and IVUS with histologic examination of cadaveric coronary arteries, measurements of intima-media thickness by OCT were more accurate than those obtained with IVUS.^34^ Indeed, the higher spatial resolution and thus sensitivity of OCT may also allow for earlier detection of CAV than IVUS. For instance, in 1 small study, intimal thickness >100 ​μm was found in 67% of vessel segments by OCT vs only 14% of the same segments by IVUS (*P* < .01).^35^ Furthermore, OCT measurements of MIT and luminal area correlate tightly with IVUS measurements and have lower interobserver variability.^36^ The Optical Coherence Tomography for Characterization of Cardiac Allograft Vasculopathy after Heart Transplantation study validated the use of OCT for early detection of CAV and proposed an intima/media ratio >1 as a cutoff for abnormal intimal thickening.^37^ More recently, 3D OCT has demonstrated significantly more paradoxical remodeling at 1 ​year after transplant than at baseline.^38^

**Figure 4:**
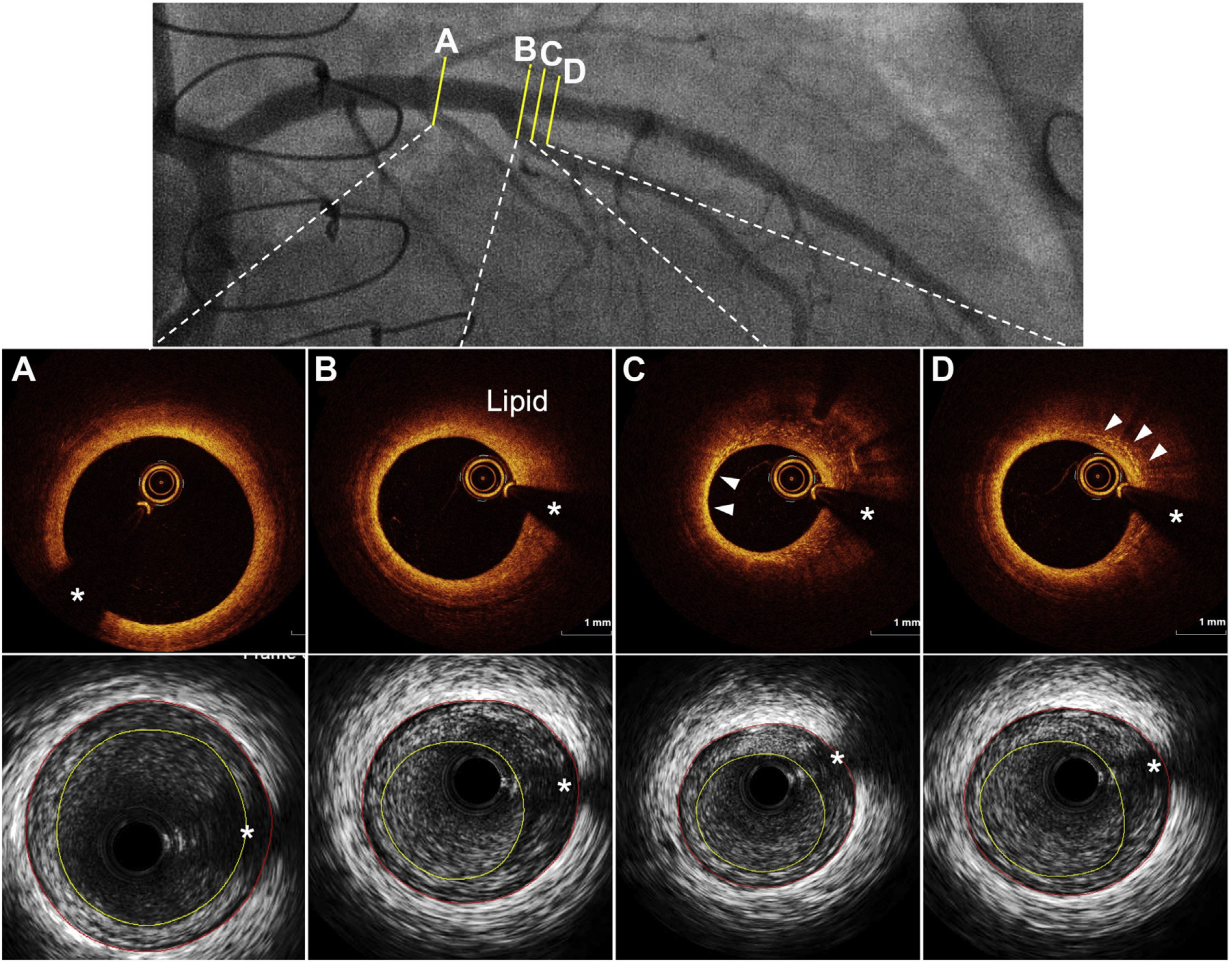
OCT and IVUS images of cardiac allograft vasculopathy. (**A**) Concentric fibrous plaque, (**B**) lipid-rich plaque, (**C**) thin-cap fibroatheroma (arrowheads), (**D**) accumulation of macrophages (arrowheads). ∗Guidewire artifact. Higher resolution of OCT offers superior delineation of each structure of plaque composition. On the other hand, IVUS can provide deeper visualization of entire vessel wall to assess plaque thickness/area and vessel remodeling. IVUS, intravascular ultrasound; OCT, optical coherence tomography.

Another advantage of OCT over IVUS is its ability to accurately distinguish the components of coronary plaques (Figure 4). A 2016 study comparing the OCT characteristics of 60 heart transplant recipients to 60 patients with native coronary atherosclerosis demonstrated that CAV lesions were more diffuse and homogenous. Transplant recipients with a history of high-grade rejection were also noted to have a significantly smaller lumen area in the distal LAD and a greater percentage of macrophage infiltration throughout the LAD than those with native atherosclerosis.^39^ Additionally, plaque characterization by OCT has provided new insights into the pathobiology of CAV. For instance, among patients farther out from their transplant, there is a significantly higher prevalence of lesions with features, detected by OCT, of typical atherosclerosis (eccentric plaque, calcification, lipid pools), vulnerable plaque (thin-cap fibroatheromas, macrophages, and microchannels), and layered complex plaque.^40^ Layered complex plaques have been attributed to repeated thrombosis followed by intimal erosion, challenging the classic paradigm that CAV is primarily due to diffuse intimal hyperplasia.^40^ Multiple studies have reported an association between microchannels and layered complex plaque, supporting the role of neovascularization in CAV.^40^, ^41^, ^42^ In a study of 45 transplant recipients, intimal neovessels were detected by OCT in 49% of the patients and were associated with a greater risk of angiographic CAV and greater MIT.^42^ Another study found that the presence of lipid pools, thin-cap fibroatheromas, macrophages, and microchannels on OCT at baseline was associated with increased plaque volume by IVUS at 1-year follow-up.^43^ Finally, several studies have demonstrated an association between a history of high-grade acute cellular rejection as well as the presence of donor-specific antibodies and abnormal OCT findings such as macrophage infiltration and intimal thickening with negative remodeling.^39^^,^^44^^,^^45^ These data support the hypothesis that immune and inflammatory-mediated processes are key factors in the development of CAV.

Additional technical advantages of OCT compared with IVUS include a faster catheter pullback speed and angiographic coregistration. Despite these features and the other aforementioned strengths, a few important limitations of OCT should be noted. First, the use of OCT may be prohibited in transplant recipients with advanced chronic kidney disease, in whom the additional injection of contrast required for coronary flushing for adequate OCT capture may risk further renal injury. However, OCT imaging of a single vessel can typically be performed with less than 15 ​mL of contrast and has been shown not to increase the risk of renal dysfunction compared with IVUS in patients with chronic kidney disease less severe than stage 5 undergoing percutaneous coronary intervention.^46^ Furthermore, most transplant patients have serial coronary angiograms, minimizing the need for test injections to find optimal angulation. Second, while OCT boasts greater spatial resolution than IVUS, its lower tissue penetration results in far field dropout and limits the assessment of plaque volume (particularly plaque containing a lipid pool or macrophage accumulation) and the intima-media interface (Figure 4).^47^ Combined OCT-IVUS catheters have been developed to address this limitation, but are not widely available for clinical use at this time and have not been studied in heart transplant recipients.^48^ Finally, in contrast to IVUS, there are no published studies to date with hard outcome data validating the prognostic impact of OCT plaque parameters in the heart transplant population.^33^ Future outcome studies, and in particular, whether OCT provides incremental outcome benefit over IVUS, will be critical in determining the adoption of OCT and its role in CAV surveillance.

### Near-infrared spectroscopy

**Figure 5:**
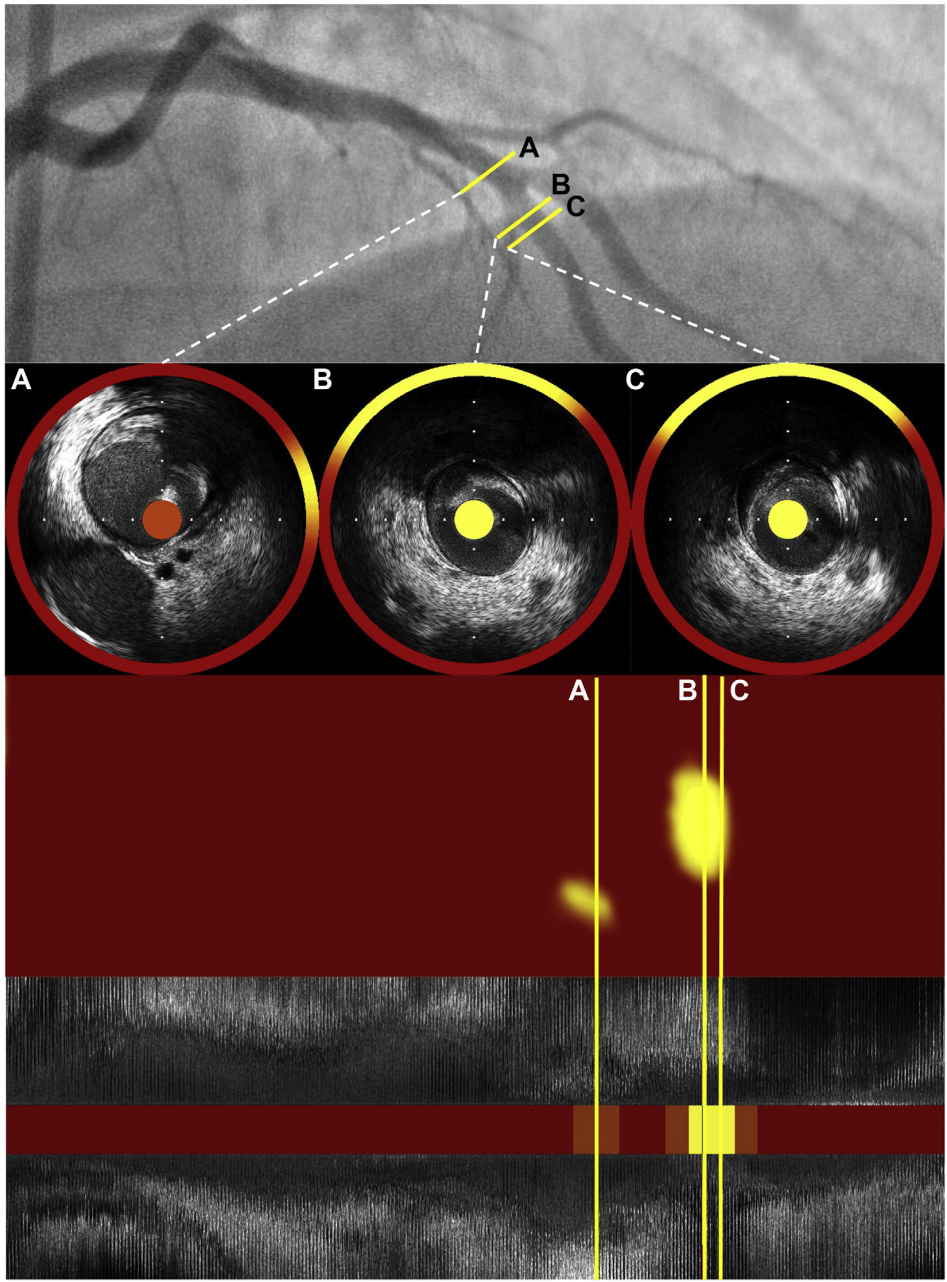
NIRS-IVUS cross-sections and NIRS chemogram. The LAD appears to have minimal disease on angiography. NIRS-IVUS reveals lipid core plaque around the mid-segment, as indicated by the longitudinal and cross-sectional yellow chemograms in panels **A**-**C**. The max LCBI_4mm_ was 326. IVUS, intravascular ultrasound; LAD, left anterior descending; NIRS, near-infrared spectroscopy.

Near-infrared spectroscopy (NIRS) is a novel intravascular imaging technology that detects the presence of lipid-rich plaque. Since its introduction in 2008, NIRS has been combined with IVUS into a single imaging catheter. Intracoronary NIRS produces a chemical pattern (chemogram) that displays the distribution of the probability of lipid-rich plaque on a color scale from red to yellow, with the x-axis indicating the location along the long axis of the vessel and the y-axis indicating the circumferential position.^49^ The lipid core burden index (LCBI) quantifies this probability of lipid-rich plaque on a scale of 0-1000 by determining the fraction of pixels indicating lipid within a region of interest, typically within a 4-mm segment (Figure 5).^49^^,^^50^ The chemogram of lipid-rich plaque identified by NIRS has been histologically validated in autopsy and in vivo studies.^51^^,^^52^

Over the past few years, NIRS has emerged as an intriguing adjunctive modality for the characterization and treatment of vulnerable lipid-rich plaque in native atherosclerosis. To date, however, there are limited data on the role of NIRS for CAV surveillance. In a study using NIRS to describe different patterns of plaque composition based on time from heart transplant, the authors reported fibrotic-predominant plaques earlier and lipid-rich predominant plaques later in the disease course.^50^ A subsequent study using NIRS to compare plaque composition of CAV in 28 heart transplant recipients (median time from transplant 4.5 ​years) with that of native atherosclerosis in 27 nontransplant patients found that transplant recipients had a greater maxLCBI_4mm_ than their counterparts in vessel segments with mild plaque burden (<40%).^49^ These findings indicated early, accelerated lipid accumulation in transplanted coronary arteries compared with native atherosclerosis. More recently, NIRS has also highlighted the role of immune factors in the development of CAV. Specifically, NIRS was used to evaluate the relationship between maxLCBI_4mm_ and prior history of high-grade acute rejection. The study found that a maxLCBI_4mm_ ​>200 distinguished these patients with a 61.5% sensitivity and 84.6% specificity.^53^

Similar to OCT, there is a lack of hard outcome data for NIRS in the CAV arena. Furthermore, despite its sensitivity for detecting lipid-rich plaques, a limitation of NIRS is its lack of depth resolution. Nonetheless, the addition of this latest technology to the invasive imaging toolbox remains promising. The decision to combine NIRS with IVUS into a single imaging catheter allows for easy adoption of this technology by operators who are already competent in performing IVUS. Moreover, given the already widespread use of IVUS for early CAV detection among heart transplant recipients, the combined imaging platform will facilitate further study of the prognostic value of NIRS-related metrics.

## Future directions

Wider adoption of these newer technologies will likely only occur following publication of outcome data and subsequent endorsement by societal guidelines (and perhaps appropriate use criteria). In addition, potentially modifiable factors that may currently contribute to underutilization of invasive imaging in heart transplant patients include the operator’s perception of inadequate time-to-reimbursement ratio and lack of education with respect to imaging interpretation. One strategy to overcome the previously described limitations of the current invasive imaging modalities and possibly drive their broader adoption has been to combine them into a single platform. For example, as described earlier, IVUS and OCT have now been integrated into a single hybrid imaging system in order to obtain the complementary benefits of both modalities, and studies to assess its clinical utility are underway.^48^^,^^54^^,^^55^ A combined OCT-NIRS imaging catheter is also under development and may provide further insights into CAV pathobiology by allowing for a more detailed analysis of plaque composition.^56^ Near-infrared autofluorescence (NIRAF) is another up-and-coming technology. NIRAF imaging is based on the principle that, in response to red-excited near-infrared light, lipid-rich plaque with necrotic core emits a greater autofluorescence detected between 700 and 900 ​nm. Of note, NIRAF has also been combined with OCT in an attempt to better characterize plaque composition.^57^

Angiography-based functional assessment of the coronary circulation is another area of growing promise for the evaluation of CAV. Coronary angiography-derived vessel fractional flow reserve (vFFR) is a new diagnostic tool that uses computational fluid dynamics to reconstruct a 3D-quantitative epicardial coronary tree and assess for functional stenosis without the need to advance a pressure wire down the vessel and administer adenosine as is traditionally done for fractional flow reserve. An initial 2020 study in heart transplant recipients demonstrated the feasibility of vFFR analysis for CAV and found that vFFR identified functional impairment in 32% of the cases where ISHLT anatomic classification did not.^58^ More recently, the same authors reported excellent correlation between vFFR and wire-based fractional flow reserve in the heart transplant population.^59^ These ongoing innovations in the realm of invasive intravascular imaging are imperative for early detection of disease, further elucidating the mechanisms of CAV, assessing effectiveness of novel therapies, and improving long-term outcomes for heart transplant recipients.

## Conclusion

Despite great progress in the field of cardiac transplantation, long-term morbidity and mortality due to CAV remain a major limitation in the field. Advances in invasive intravascular imaging have provided greater sensitivity and thus earlier diagnosis of CAV, leading to the emergence of IVUS as the standard of care for CAV screening today. More recently, the addition of OCT and NIRS to the invasive imaging armamentarium has allowed for more detailed plaque characterization and, in doing so, shed further light on possible mechanisms of CAV. The salient strengths and limitations of these imaging platforms are summarized in the Central Illustration. Future studies evaluating the prognostic impact of parameters from these established and emerging invasive imaging modalities in the current era of heart transplantation are needed to determine their ultimate role in the contemporary CAV screening paradigm.

**Central Illustration:**
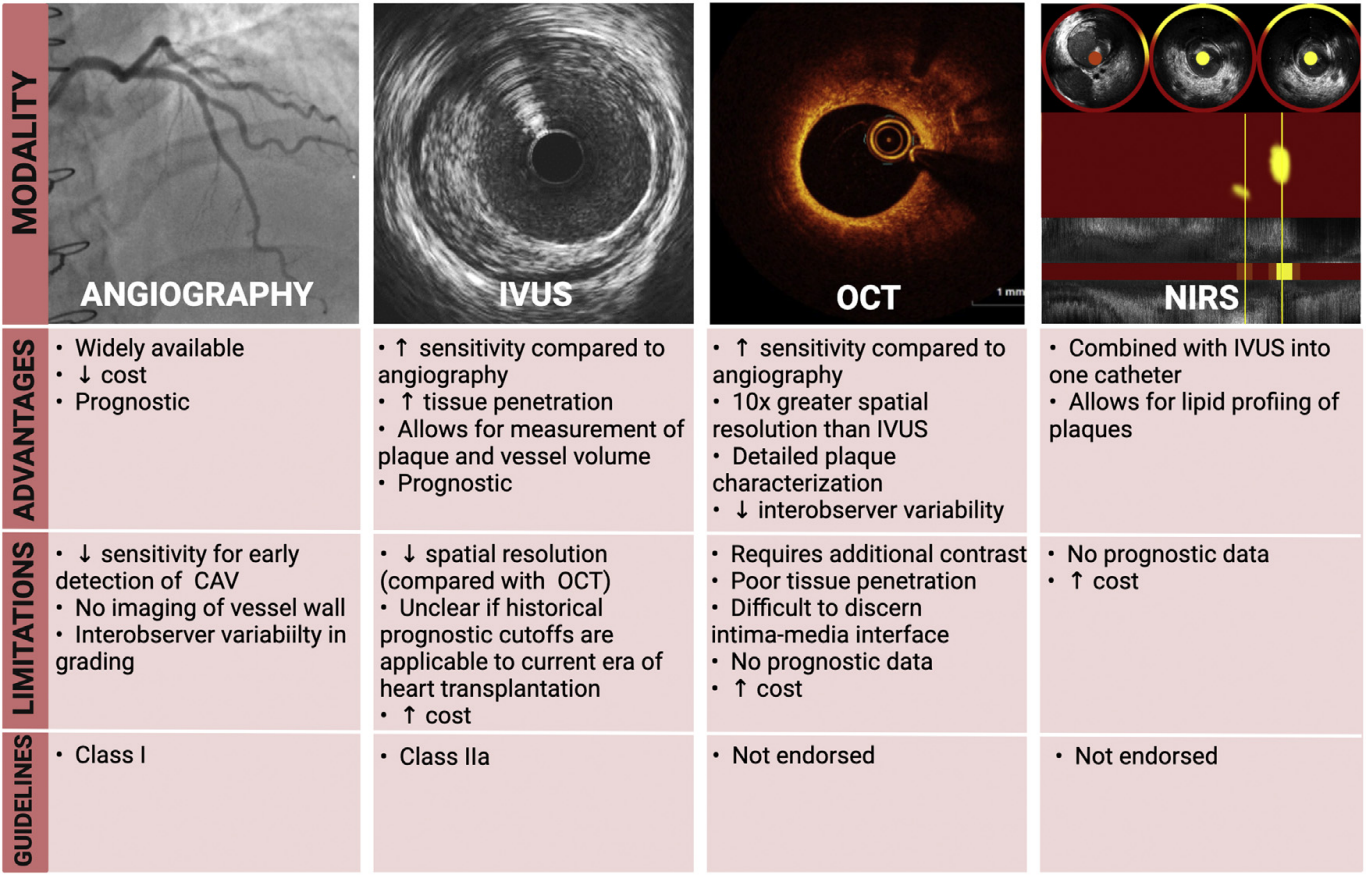
Invasive imaging modalities for diagnosing CAV. Created with BioRender.

## Declaration of competing interest

Dr Ali receives institutional research support from 10.13039/100011949Abbott Vascular and 10.13039/100016476Cardiovascular Systems Inc and consulting fees from Amgen, AstraZeneca, and Boston Scientific and has equity in Shockwave Medical. Dr Fearon receives institutional research support from 10.13039/100011949Abbott Vascular, 10.13039/100008497Boston Scientific, and 10.13039/100004374Medtronic. Dr Parikh receives research support from the 10.13039/100000968American Heart Association, 10.13039/100005565Janssen, 10.13039/100018503InfraRedx, and 10.13039/100011949Abbott Vascular and consulting fees from Abbott Vascular. The remaining authors report that they have no relationships with industry relevant to the contents of this manuscript to disclose.
